# Co- and Post-Treatment with Lysine Protects Primary Fish Enterocytes against Cu-Induced Oxidative Damage

**DOI:** 10.1371/journal.pone.0147408

**Published:** 2016-01-26

**Authors:** Xue-Yin Li, Yang Liu, Wei-Dan Jiang, Jun Jiang, Pei Wu, Juan Zhao, Sheng-Yao Kuang, Ling Tang, Wu-Neng Tang, Yong-An Zhang, Xiao-Qiu Zhou, Lin Feng

**Affiliations:** 1 Animal Nutrition Institute, Sichuan Agricultural University, Chengdu, Sichuan, China; 2 Fish Nutrition and Safety Production University Key Laboratory of Sichuan Province, Sichuan Agricultural University, Chengdu, Sichuan, China; 3 Key Laboratory for Animal Disease-Resistance Nutrition of China Ministry of Education, Sichuan Agricultural University, Chengdu, Sichuan, China; 4 Animal Nutrition Institute, Sichuan Academy of Animal Science, Chengdu, 610066, Sichuan, China; 5 Institute of Hydrobiology, Chinese Academy of Sciences, Wuhan, 430072, China; University of Florida, UNITED STATES

## Abstract

The aim of the work was primarily to explore the protective activity pathways of lysine against oxidative damage in fish *in vivo* and in enterocytes *in vitro*. First, grass carp were fed diets containing six graded levels of lysine (7.1–19.6 g kg^-1^ diet) for 56 days. Second, the enterocytes were treated with different concentrations of lysine (0–300 mg/L in media) prior to (pre-treatment), along with (co-treatment) or following (post-treatment) with 6 mg/L of Cu for 24 h. The results indicated that lysine improved grass carp growth performance. Meanwhile, lysine ameliorated lipid and protein oxidation by elevating the gene expression and activity of antioxidant enzymes (superoxide dismutase (SOD), glutathioneperoxidase (GPx), glutathione-S-transferase (GST) and reductase (GR)), and nuclear factor erythroid 2-related factor 2 (Nrf2) mRNA levels in fish intestine. The *in vitro* studies showed that co- and post-treatment with lysine conferred significant protection against Cu-induced oxidative damage in fish primary enterocytes as measured by 3-(4,5-dimethylthiazol-2-yl)-2,5-diphenyltetrazolium bromide (MTT) OD values, along with alkaline phosphatase (ALP) and lactate dehydrogenase activities, and the depletion of protein carbonyl (PC), malondialdehyde (MDA) and 8-hydroxydeoxyguanosine contents. Moreover, lysine co-treatment decreased the activities and mRNA level of cellular SOD, GPx, GST and GR compared with the Cu-only exposed group. Gene expression of the signalling molecule Nrf2 showed the same pattern as that of SOD activity, whereas Kelch-like ECH-associated protein 1b (Keap1b) followed the opposite trend, indicating that co-treatment with lysine induced antioxidant enzymes that protected against oxidative stress through Nrf2 pathway. In addition, post-treatment with lysine increased proteasomal activity and blocked the Cu-stimulated increase in mRNA levels of GST and associated catalase (CAT) and GST activities (*P*<0.01 and *P*<0.001). GR activity and gene expression, and glutathione (GSH) content followed an opposite trend to GST activity (*P*<0.05). Thus, post-treatment of lysine elevated protein and DNA repair abilities and ameliorated the cellular redox state of enterocytes. The overall results suggest that lysine plays a significant role in the protection of fish intestine *in vivo* and *in vitro* through the induction of key antioxidant protection.

## Introduction

Lysine is an indispensable component of the fish diet [1]. Our previous study indicated that dietary lysine could improve digestive enzyme and brush-border membrane enzyme activities of sub-adult grass carp [2]. The regulation of enzyme activity is related to gene expression [3], which is partially controlled by nutritional factors in fish [4]. However, very few reports have examined the effects of lysine on the mRNA abundance of digestive enzymes and brush border membrane enzymes in fish. Studies have shown that intestinal enzyme protein synthesis is regulated by the target of rapamycin (TOR) signalling pathway [4]. In Atlantic salmon (*salmo salar*), high levels of lysine intake upregulated IGF-1 mRNA levels in muscle [5]. Moreover, IGF-1 regulated downstream mTOR activation in muscle cells [6]. However, whether lysine regulates gene expression of digestive and absorptive enzymes by modulating TOR signalling in fish has not yet been studied.

The normal function of fish digestive organs depends on the structural integrity of the enterocytes, which can be affected by the antioxidant capacity [7,8]. Our previous studies have shown that dietary lysine enhances fish intestinal antioxidant ability in part by increasing GSH levels and the activities of antioxidant enzymes [2]. However, the signalling pathway and underlying mechanism have not yet been described in fish. *In vivo*, three pathways are generally used to describe the protective effects of nutrients: prevention, interception and repair [9]. The prevention of reactive oxygen species (ROS) formation is the first line of defence against oxidative insult [10]. Once formed, free radicals trigger damage by binding to the biomacromolecules of the cell [11]. Interceptive efficacy can deactivate radical compounds and reactive metals for further toxicity [12]. In addition, protection against oxidant effects can also occur by damage repair once it has appeared [13]. We have previously shown that myo-inositol could protect fish intestinal epithelial cells against Cu-induced oxidative injury by the three pathways mentioned above [14,15]. However, the antioxidant pathways may vary among the different nutrients. In vertebrates, micronutrients primarily exhibit their antioxidant efficiency by metal chelation or electron donation [16,17]. However, several amino acids have demonstrated their antioxidant efficiency by increasing cellular GSH content and antioxidant enzyme activities [18]. In rainbow trout, lysine is catabolised to glutamate by the saccharopine pathway [19]. Stoll et al. [20] reported that enteral glutamate can be converted into GSH in piglets. Moreover, the lysine residue is one of the major sites of damage during radical attack, thus lysine supplements may provide alternate targets for free radicals, resulting in the stabilization of proteins *in vitro* [21]. Therefore, we sought to investigate whether lysine might protect the intestine against oxidative damage through prevention, interception or repair pathways.

Antioxidant enzyme activities are partially dependent on their gene transcription via the Nrf2 complex in vertebrates [22,23]. Our laboratory recently cloned Nrf2 (GenBank accession number KF733814), Keap1a (GenBank accession number KF811013) and Keap1b (GenBank accession number KJ729125) cDNA from grass carp. Nutrients may modulate Nrf2 gene expression differently. Studies from our lab have shown that dietary leucine up-regulated the mRNA abundance of Nrf2 in intestine of grass carp [24]. However, in kidney, dietary choline decreased Nrf2 m-RNA levels of juvenile Jian carp (*Cyprinus carpio* var. Jian) [25]. Zhang and Hannink [26] reported that Keap1 is a major regulator of Nrf2 stability by targeting the Neh2 domain of Nrf2 for ubiquitination. Further studies indicated that seven lysine residues within the Neh2 domain are the acceptor sites for Keap1-targeted ubiquitination and are critical determinants of Nrf2 stability [27]. These data indicated that lysine might modulate Nrf2 signalling molecules in fish, which should be investigated.

Grass carp (*Ctenopharyngodon idella*) is an economically important food that is farmed in China [28]. Thus, there is an urgent need to develop a nutrient-balanced practical diet for grass carp. Moreover, the inclusion of optimised lysine amounts is required to formulate cost-effective fish feed [29]. Our previous study estimated a lysine requirement for grass carp 450–1000 g in weight [2], but the nutrient requirements vary with the growth stage of the fish [30]. Therefore, it is important to determine the optimal dietary lysine requirement for grass carp 250–500 g in weight.

The present study investigated the protective effects of lysine on fish intestine function and integrity. We first established the effect of dietary lysine on growth, digestive capacity and absorptive function, as well as on intestinal antioxidant ability. We then assessed the preventive, interceptive and repair abilities of lysine in a copper (Cu)-exposed primary enterocyte model. Our findings offer preliminary evidence for the role of lysine in protecting against intestinal oxidative injury in fish.

## Materials and Methods

### 2.1 Chemicals

Fish meal was obtained from Pesquera Lota Protein Ltd. (Villagram, Chile). Casein and gelatin were obtained from Hulunbeier Sanyuan Milk Co., Ltd. (Inner Mongolia, China) and Rousselot Gelatin Co., Ltd. (Guangdong, China). Crystalline amino acids were obtained from Yimengsi Ltd. (Shanghai, China). L-Lysine, collagenase, dispase, insulin, transferrin, copper sulphate pentahydrate (CuSO_4_·5H_2_O), streptomycin sulphate, benzylpenicillin, triton X-100, dimethyl sulphoxide (DMSO) and 3-(4,5-dimethylthiazol-2-yl)-2,5-diphenyltetrazolium bromide (MTT) were obtained from Sigma (St. Louis, MO, USA). Hank’s balanced salt solution (HBSS), fetal bovine serum (FBS) and Dulbecco’s Modified Eagle’s Media (DMEM) were purchased from Hyclone (Logan, UT, USA). DMEM (lysine-free) was obtained from Beijing Tsing Skywing Bio. Tech. Co. Ltd. (Beijing, China).

### 2.2 Fish and treatment (*in vivo*)

The experimental designs and procedures were all approved by the Animal Care and Use Committee (IACUC) of Sichuan Agricultural University. Young grass carp were obtained from commercial cultivation (Bailong Lake, Sichuan, China) and conditioned for 14 days. A total of 720 fish (254.9 ± 1.1 g) were randomly distributed into 24 cages (1.4×1.4×1.4 m). The components and nutritional parameters of the basal diets are shown in S1 Table. Crystalline L-lysine was added in increments of 2.5 g kg^-1^ to the diet. The diets were kept in an isonitrogenous state by reducing the glycine levels to compensate for the increasing lysine levels. The pH values of all of the diets were maintained at 7.0 through the addition of NaOH (6.0 N) [31]. The pellets were stored at -20°C until use [32]. The lysine concentration in each diet was measured by using HPLC (Agilent Technologies, Palo Alto, CA, USA). The fish were fed four times a day with their respective diets for 56 days. During the experimental period, fish were raised under natural light conditions. Dissolved oxygen and pH were maintained at 7.0 ± 0.5 mg L^-1^ and 7.5 ± 0.3, respectively. The water temperature was 25 ± 2°C.

### 2.3 Cell isolation and culture (*in vitro*)

Primary enterocyte culture was performed following a method previously described by Jiang et al. [33] with some modification. The foregut and midgut from healthy grass carp (50–100 g) were rapidly removed from the carcass and then opened and rinsed in Hank’s balanced salt solution (HBSS) containing 100 IU/mL benzylpenicillin and 100 μg/mL streptomycin sulphate. Enterocytes were dissociated enzymatically (collagenase and dispase). Cells were plated at a density of 1× 10^5^ viable cells per millilitre into 24-well collagen-coated microplates for viability and enzyme activity assays, or 5 × 10^6^ viable cells per millilitre into six-well collagen-coated microplates for gene expression and enzyme-linked immunosorbent (ELISA) assay. The cells were suspended in modified DMEM medium containing 5% foetal calf serum (FBS), 0.02 mg/mL transferrin, 0.01 mg/mL insulin, 100 IU/mL benzyl penicillin and 100 μg/mL streptomycin sulphate. The cultures were allowed to attach and grow at 26 ± 0.5°C for 72 h. On the following day, the cells were incubated in different media of lysine-free DMEM containing different concentrations of lysine (0, 60, 120, 180, 240 and 300 mg L^-1^) or absolute Cu (6 mg L^-1^). The optimal cytotoxic dose of Cu (6 mg L^-1^ for 24 h) for fish primary enterocytes was established in our pilot studies based on cell viability assays results and oxidative damage indices, following the method of Jiang et al. [14] (data not shown). Three treatment regimens were used: (a) cells were cultured in various concentrations of lysine for 72 h prior to the 24 h Cu insult (pre-treatment group), (b) cells were cultured in various concentrations of lysine along with the Cu insult for 24 h (co-treatment group) and (c) cells were cultured in various concentrations of lysine for 72 h following 24 h of Cu insult (post-treatment group). Four wells were used for each treatment. After the indicated time of incubation, the supernatants and the cells were collected.

The media were collected for determination of the lactic dehydrogenases (LDH) activity and free Cu^2+^ ion concentration (co-treatment regimen) as described by Jiang et al. [15]. For oxidative damage indices and enzymatic activities analysis, the enterocytes were collected and homogenised in an ice-cold solution (pH 7.4) containing 0.01 mol/L Tris-HCl, 0.01 mol/L sucrose, 0.0001 mol/L EDTA-2Na, 0.75% NaCl and 0.05% Triton X-100, as described by Chen et al. [34]. For gene expression analysis, the enterocytes were collected, and total RNA was extracted using the RNA prep Pure Cell Kit (Tiangen, Beijing, China) per the manufacturer’s instructions.

### 2.4 Analysis and measurement

#### 2.4.1 Determination of growth parameters (*in vivo*)

Growth performance and nutrient utilisation of grass carp after the 56 day feeding trial were determined using the following formulae:

Percentage weight gain(PWG)(%)=100×(FBW-IBW)/IBW

Feed conversion ratio(FCR)=FI/(FBW-IBW)

SGR(%/day)=100×(ln FBW - ln IBW)/per number of days,

where IBW is the initial weight, FBW is the final weight and FI is the feed intake (g).

#### 2.4.2 Cell viability, alkaline phosphatase and lactic dehydrogenases assays (*in vitro*)

Analysis of enterocyte viability was performed by an MTT assay as described by Jiang et al. [14]. ALP activity was assayed according to the method described by Wergedal and Baylink [35]. Protein concentration was determined by the Coomassie blue method as described by Bradford [36]. Membrane integrity assays were measured using the lactate dehydrogenase (LDH) release assay following the method as described by Jiang et al. [15].

#### 2.4.3 Antioxidant status *in vivo* and *in vitro*

At the end of the experiment, fish from each cage were starved for 12 h [37] and then anaesthetised in a benzocaine bath (50 mg/L) as described by Bohne et al. [32]. The tissue samples of intestine were homogenised in an ice-cold physiological saline solution (1:10, w/v). The homogenates were then centrifuged at 6000 × *g* and 4°C for 20 min. Supernatants were then transferred to new tubes and stored at -80°C until analysis. For each enzyme activity assay, assay dilution tests were performed first to ensure the optimal ratio between enzyme and substrate. Malondialdehyde (MDA) and protein carbonyl (PC) contents were analysed according to Livingstone et al. [38] and Armenteros et al. [39], respectively. Reduced GSH was quantitated by the method of Vardi et al. [40]. The activities of superoxide dismutase (SOD) and glutathioneperoxidase (GPx) were measured according to Vardi et al. [40]. The activities of catalase (CAT) and glutathione-S-transferase (GST) were determined using the methods as described by Aebi and Lushchak et al. [41,42]. Glutathione reductase (GR) activity was measured according to Lora et al. [43]. The level of enterocyte 4-hydroxy-2-nonenal (4HNE), 8-hydroxydeoxyguanosine (8-OHdG) and proteasomal activities were measured by using the corresponding ELISA (Chenglin, Beijing, China) kit according to the manufacturer’s instructions.

#### 2.4.4 Real-time PCR analysis

The total RNA of intestine and enterocytes was extracted using the RNAiso Plus Kit (Takara, Dalian, China) and the RNAprep Pure Cell Kit (Tiangen, Beijing, China), respectively, as per the manufacturer’s instructions. The yield and quality of RNA were assessed using electrophoresis in a 1% agarose gel and photographed using a UV transilluminator. Specific primers and optimal annealing temperatures are presented in S2 Table. The qRT-PCR was performed with the SYBR^®^ Prime Script qRT-PCR Kit Ⅱ (Takara, Dalian, Shandong, China) according to the manufacturer’s instructions. The results were analysed using the 2^−ΔΔCT^ method after verification that the primers amplified with an efficiency of approximately 100% [44]. According to the results of our preliminary experiment on the evaluation of internal control genes (data not shown), β-actin was used as a reference gene to normalise cDNA loading. The amplification efficiency of the housekeeping and target genes was calculated by the standard curves of the specific gene generated from 10-fold serial dilutions.

### 2.5 Statistical analysis

All of the data are presented as the mean ± standard error (SE) and were analysed by ANOVA. Duncan's multiple range tests were used to assess differences between treatment groups at the level of *P*<0.05 (*in vivo*). The data were analysed by the student’s t-test, and *P<0.05, **P<0.01 and ***P<0.001 were used as the criteria for significance (*in vitro*). Statistical analyses were carried out using SPSS 17.0 (SPSS Inc., Chicago, IL, USA).

## Results

### 3.1 Growth performance

**Fig 1:**
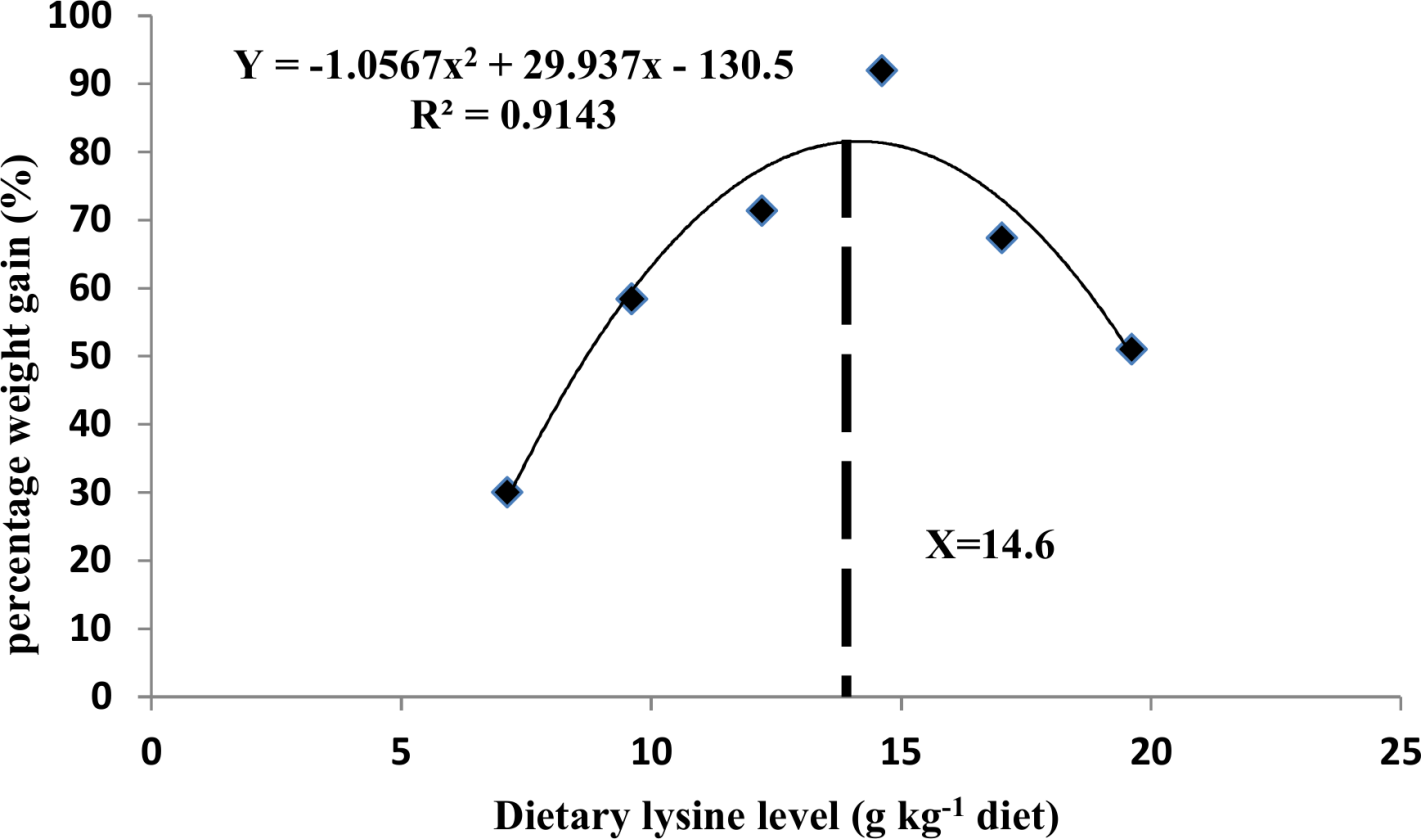
Quadratic regression analysis of the relationship between PWG (percentage weight gain) and dietary lysine level of grass carp.

The effects of graded levels of dietary lysine on growth performance are shown in Table 1. Fish fed a diet with 7.1 g kg^-1^ lysine showed the lowest FBW and SGR (*P*<0.05). The FI gradually increased with lysine levels up to 14.6 g kg^-1^ diet and then decreased (*P*<0.05). The FCR was also affected by dietary lysine, exhibiting an opposite trend to the FI. The estimation of lysine requirement by the quadratic regression analysis based on PWG was 14.2 g kg^-1^ (47.3 g kg^-1^ of dietary protein) (Fig 1).

**Table 1:** Growth parameters of grass carp (*Ctenopharyngodon idella*) fed the experiment diets for 56 days^1^.

	Dietary lysine levels (g kg^-1^ diet)					
	7.1	9.6	12.2	14.6	17.0	19.6
IBW	254.8±0.2	254.6±0.6	255.7±0.4	254.5±0.7	254.7±0.4	255.4±0.2
FBW	331.4±4.3^a^	403.5±4.5^c^	438.3±4.3^d^	488.6±3.3^e^	426.4±5.8^d^	385.9±5.6^b^
PWG	30.1±1.7^a^	58.5±1.7^c^	71.4±1.5^d^	92.0±1.7^e^	67.4±2.4^d^	51.1±2.2^b^
SGR	0.55±0.03^a^	0.96±0.02^c^	1.12±0.02^d^	1.36±0.02^e^	1.07±0.03^d^	0.86±0.03^b^
FI	238.3±4.4^a^	343.8±10.2^c^	367.4±4.9^d^	421.3±11.6^e^	366.7±5.1^d^	305.4±3.0^b^
FCR	3.14±0.14^d^	2.31±0.07^c^	2.02±0.06^ab^	1.80±0.06^a^	2.14±0.07^bc^	2.35±0.09^c^

^1^Results are the means ± SE (n = 4). Different letter within the same row denoted significant differences (*P*<0.05), with Duncan's multiple range tests.

### 3.2 Gene expression of digestive and brush border enzymes and TOR-related signalling molecules

The effects of lysine on intestinal digestive enzyme and brush border enzyme gene expression are shown in S3 Table. The gene expression of trypsinogen 1 was higher in fish fed 12.2 and 14.6 g lysine kg^-1^ compared with any other group (*P*<0.05). The chymotrypsinogen mRNA levels showed the highest values in diets containing 9.6 g lysine kg^-1^, and the lowest in diets containing 19.6 g lysine kg^-1^ (*P*<0.05). However, no significant differences were found in amylase gene expression among all of the groups (*P*>0.05). The mRNA abundance of the Na^+^/K^+^-ATPase improved with increasing lysine levels up to 12.2 g kg^-1^ and decreased thereafter in the intestinal segments (*P*<0.05). CK gene expression in MI was higher in fish fed 14.6–19.6 g lysine kg^-1^ than in fish fed 7.1–12.2 g lysine kg^-1^ (*P*<0.05). However, the CK mRNA levels in PI and DI were not affected by dietary lysine concentrations (*P*>0.05). The TOR m-RNA levels in PI, MI and DI were improved with increasing dietary lysine levels up to 17.0, 14.6 and 12.2 g kg^-1^, and decreased thereafter (*P*<0.05). Gene expression of 4E-BPs in DI showed the opposite trend to the TOR m-RNA levels, with the lowest values observed in fish fed the 12.2 g lysine kg^-1^. However, no significant differences were observed in the mRNA abundance of the 4E-BPs in PI and MI (*P*>0.05).

### 3.3 Intestinal protein oxidants, lipid peroxidation and antioxidant parameters

As shown in Table 2, malondialdehyde (MDA) and protein carbonyl (PC) contents were negatively affected by dietary lysine levels, with the lowest values obtained for fish fed a 14.6 g lysine kg^-1^ diet (*P*<0.05). The activities of CAT and GR were elevated with dietary lysine levels up to 17.0 g kg^-1^ and then significantly decreased (*P*<0.05). The activity of GST was gradually increased with increasing dietary lysine, with the highest values obtained at 14.6 g lysine kg^-1^. SOD and GPx activities improved by increasing the dietary lysine levels up to 9.6 g kg^-1^ and decreased significantly above this dose (*P*<0.05). GSH content showed the same trend as the SOD activity (*P*<0.05).

**Table 2:** Malondialdehyde content (MDA, nmol/mg prot), protein carbonyl content (PC, nmol/mg prot), superoxide dismutase (SOD, U/mg prot), catalase (CAT, U/mg prot), glutathione-S-transferase (GST, U/mg prot), glutathione peroxidase (GPX, U/mg prot), glutathione reducase (GR, U/g prot) activities and glutathione (GSH, mg/g prot) content in intestine of fish fed the experimental diets for 56 days^1^.

	Dietary lysine levels (g kg^-1^ diet)					
	7.1	9.6	12.2	14.6	17.0	19.6
MDA	5.44±0.15^e^	4.26±0.07^d^	2.89±0.12^b^	2.30±0.05^a^	3.61±0.11^c^	4.28±0.10^d^
PC	5.77±0.24^b^	4.59±0.18^a^	4.86±0.19^a^	4.34±0.09^a^	6.18±0.20^b^	6.15±0.23^b^
SOD	29.88±0.82^b^	33.59±0.95^c^	25.91±1.01^a^	26.94±0.87^a^	26.61±1.07^a^	25.44±1.00^a^
CAT	43.30±1.20^b^	42.91±1.70^b^	43.53±1.26^b^	53.18±2.18^c^	55.31±2.17^c^	33.77±0.70^a^
GST	10.84±0.31^ab^	10.09±0.27^a^	10.51±0.29^ab^	20.49±0.55^d^	13.45±0.55^c^	11.70±0.50^b^
GPx	124.84±5.35^b^	141.63±5.44^c^	110.54±1.85^a^	103.05±3.54^a^	104.84±3.92^a^	105.63±4.41^a^
GR	66.97±2.02^a^	72.21±2.16^ab^	71.41±2.00^ab^	77.31±2.69^b^	121.96±3.98^d^	89.17±2.76^c^
GSH	1.05±0.05^d^	2.51±0.06^e^	1.01±0.03^d^	0.81±0.04^c^	0.60±0.03^b^	0.39±0.02^a^

^1^ Results are the means ± SE (n = 5). Different letter within the same row denoted significant differences (*P*<0.05), with Duncan's multiple range tests.

### 3.4 Antioxidant-related gene expression in fish intestine

The intestinal gene expression levels of CAT, SOD, GST, GPx, GR, Nrf2, Keap1a and Keap1b in fish fed dietary graded concentrations of lysine are shown in S1 Fig. The expression levels of SOD, GPx and GST concomitantly elevated with lysine levels up to 14.6 g lysine kg^-1^ (*P*<0.05) and then significantly decreased. The m-RNA level of CAT and Nrf2 significantly increased when diet lysine levels increased to 9.6 and 12.2 g kg^-1^ and then decreased (*P*<0.05). GR and Keap1b gene expression showed an opposite pattern of CAT, with the lowest mRNA levels observed in fish that received 9.6 and 14.6 g lysine kg^-1^, respectively (*P*<0.05). However, the Keap1a mRNA level was not changed by the dietary lysine level (*P*>0.05).

### 3.5 Prevention, interception and repair effects of lysine in fish enterocytes

#### 3.5.1 Effect of pre-, co- and post-treatment with lysine on MTT OD values, LDH and ALP activities

**Fig 2:**
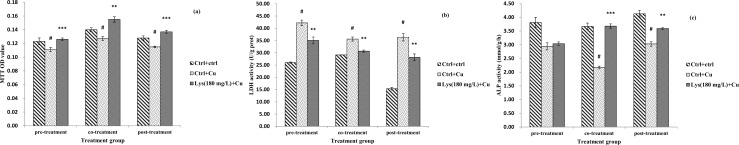
Effects of pre-, co- and post-treatment of lysine on the LDH activity (U/g prot), MTT OD and ALP activity (mmol of nitrophenol released g^-1^ tissue h^-1^) of fish primary enterocytes cells. The data represent the means±S.E. of four replicates. # indicated significant difference compared with control values, and *P<0.05, **P<0.01, and ***P<0.001 indicated significant difference from the 6 mg L^-1^ Cu alone group, with Student’s t-test. LDH, lactic acid dehydrogenase; MTT, 3-(4, 5-dimethylthiazol-2-yl)-2,5-diphenyltetrazolium bromide; ALP, alkaline phosphatase.

The protective potential of lysine against the toxic insult of Cu (6 mg L^-1^) in fish enterocytes was assessed by MTT OD values and by determination of LDH and ALP activities (Fig 2 and S4 Table). Compared with the control, enterocytes receiving 6 mg L^-1^ Cu exhibited a significant decrease in MTT OD values accompanied by an increase in LDH leakage (*P*<0.05). A dose-dependent increase in the MTT OD values was evident in pre-, co- and post-treatments with lysine (*P*<0.05, *P*<0.01 and *P*<0.001). The opposite trend in LDH activities was modestly observed in the pre-, co- and post-treatment groups. Compared with the control, ALP activities decreased in the group that was exposed to Cu only (*P*<0.05). In the co- and post-treatment regimens, there were significant increases in ALP activity in media containing ≥ 120 and ≥180 mg lysine L^-1^, respectively (*P*<0.001, *P*<0.01 and *P*<0.05). However, none of the lysine doses had any effect on ALP activity in the pre-treatment regimen after Cu exposure (*P*>0.05).

#### 3.5.2 Effect of pre-, co- and post-treatment with lysine on lipid peroxidation, protein oxidation and DNA damage

**Fig 3:**
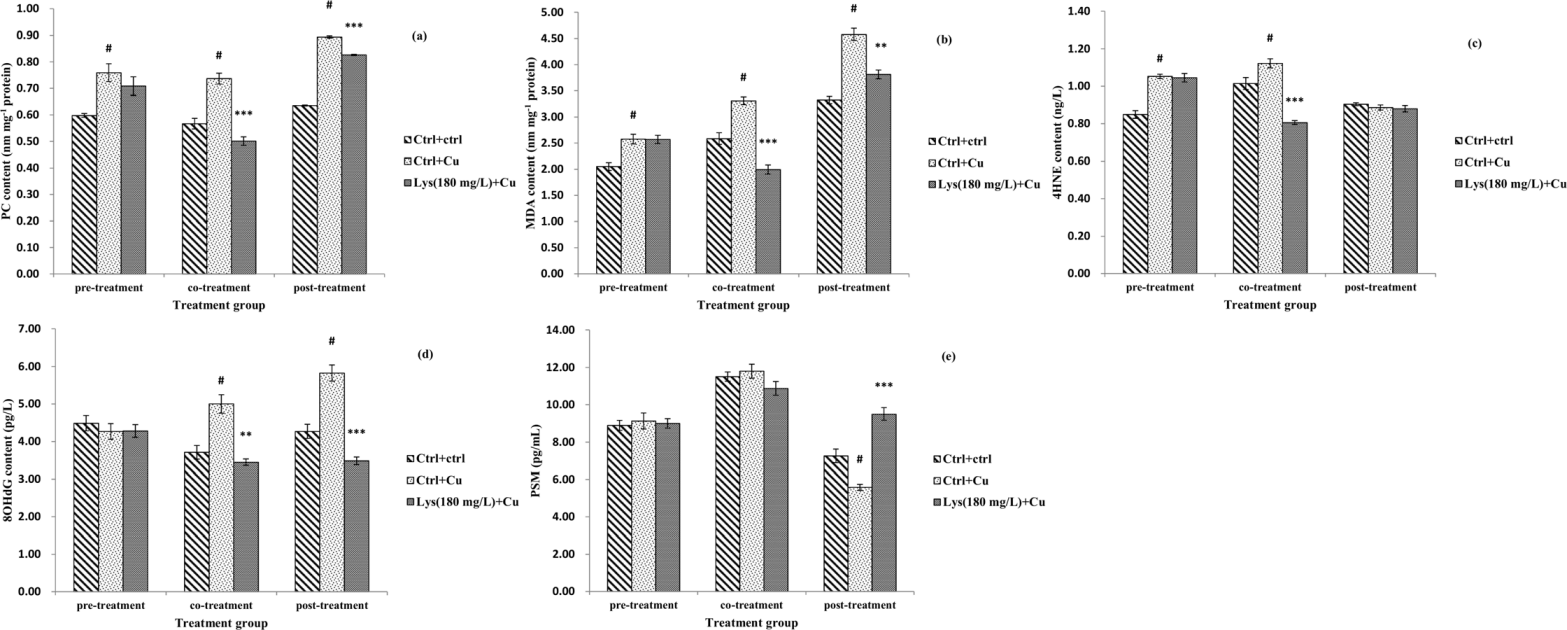
Effects of pre-, co- and post-treatment of lysine on the PC content (nm mg^-1^ protein), MDA content (nm mg^-1^ protein), 4HNE content (ng L^-1^), 8OHdG content (ng L^-1^) and PSM activity (pg ml^-1^) of fish primary enterocytes cells. The data represent the means±S.E. of four replicates. # indicated significant difference compared with control values, and *P<0.05, **P<0.01, and ***P<0.001 indicated significant difference from the 6 mg L^-1^ Cu alone group, with Student’s t-test. PC, protein carbonyl; MDA, malondialdehyde content; 4HNE, 4-Hydroxy-2-nonenal; 8OHdG, 8-hydroxydeoxyguanosine; PSM, proteasomes.

The results in Fig 3 indicate that the PC and MDA content were increased in the Cu-only exposed group compared with the control (*P*<0.05). In the co- and post-treatment regimens, 180 mg lysine L^-1^ media decreased MDA and PC levels (*P*<0.01 and *P*<0.001). Cu-exposure induced an increase in 4-hydroxy-2-nonenal (4HNE) content in the pre- and co-treatment regimens (*P*<0.05), but could only decrease the 4HNE content when co-treated with lysine (*P*<0.001). In the co- and post-treatment regimens, 8-hydroxydeoxyguanosine (8OHdG) content increased in the Cu-only exposed group, but the values significantly decreased with lysine supplementation (*P*<0.01 and *P*<0.001). In the post-treatment regimen, the proteasomal (PSM) activity was decreased in the Cu-only exposed group (*P*<0.05), but post-treatment with lysine elevated the PSM activity (*P*<0.001). However, no significant change in the PC, MDA or 8OHdG contents was observed in the pre-treatment regimen or in the PSM activity under pre- and co-treatment conditions (*P*>0.05).

#### 3.5.3 Effect of pre-, co- and post-treatment with lysine on antioxidant-related factors

**Fig 4:**
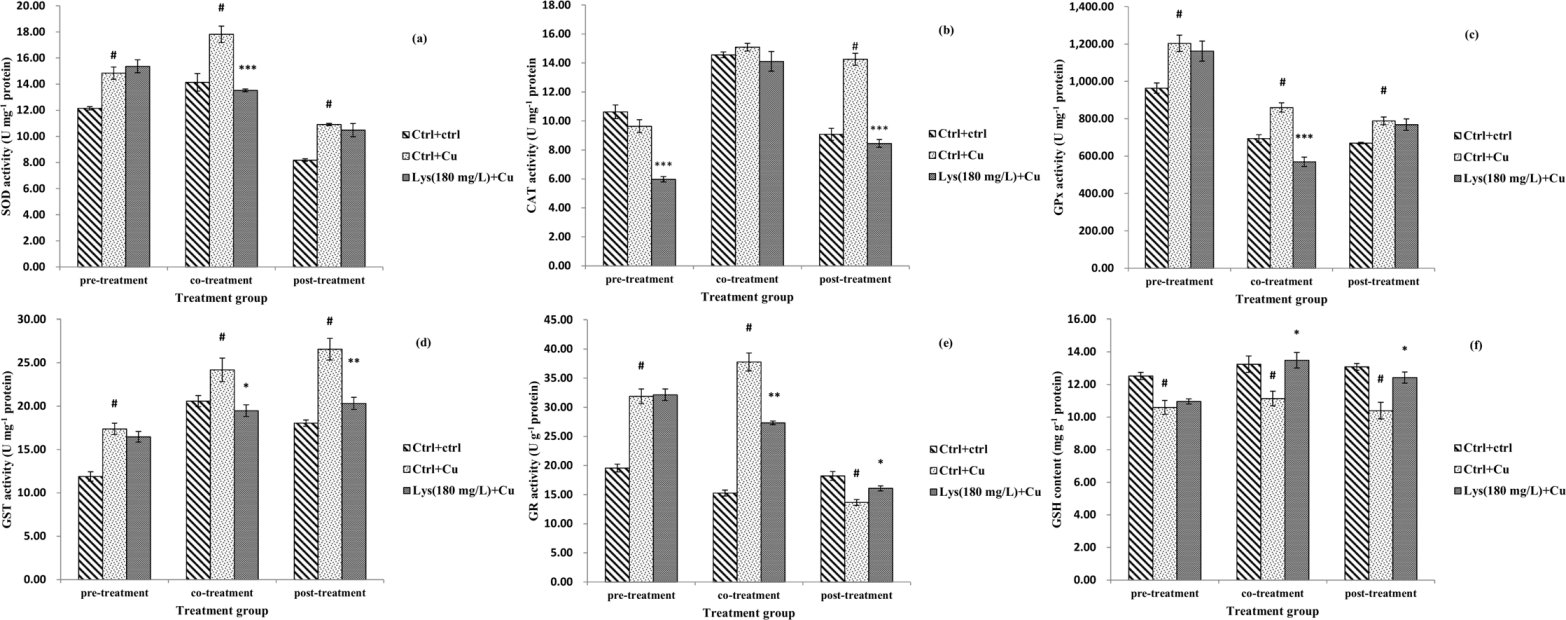
Effects of pre-, co- and post-treatment of lysine on the activity of SOD (U/mg prot), CAT (U/mg prot), GPx (U/mg prot), GST (U/mg prot) and GR (U/g prot), and GSH (mg/g prot) content of fish primary enterocytes cells. The data represent the means±S.E. of four replicates. # indicated significant difference compared with control values, and *P<0.05, **P<0.01, and ***P<0.001 indicated significant difference from the 6 mg L^-1^ Cu alone group, with Student’s t-test. SOD, copper/zinc superoxide dismutase; CAT, catalase; GPx, glutathione peroxidase; GST, glutathione-S-transferase; GR, glutathione reducase enzymes; GSH, glutathione.

**Fig 5:**
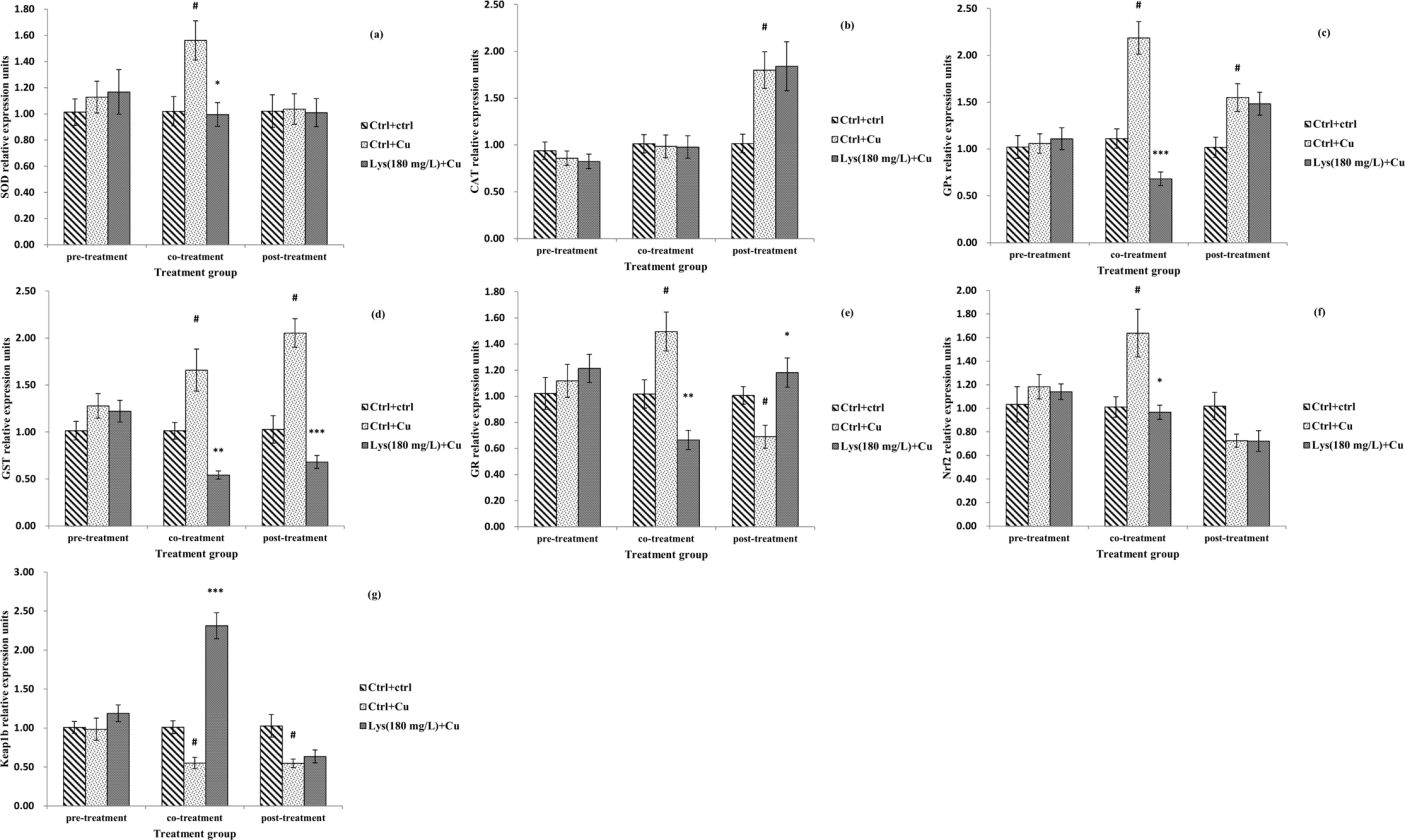
Effects of pre-, co- and post-treatment of lysine on the gene expression of SOD, CAT, GPx, GST, GR, Nrf2 and Keap1b of fish primary enterocytes cells. The data represent the means±S.E. of four replicates. # indicated significant difference compared with control values, and *P<0.05, **P<0.01, and ***P<0.001 indicated significant difference from the 6 mg L^-1^ Cu alone group, with Student’s t-test. SOD, copper/zinc superoxide dismutase; CAT, catalase; GPx, glutathione peroxidase; GST, glutathione-S-transferase; GR, glutathione reducase enzymes; Nrf2, NF-E2-related factor-2; Keap1b, Kelch-like ECH-associated protein 1b.

Antioxidant enzyme activity and transcriptional levels of Nrf2 and related signalling molecules were also evaluated (Fig 4 and Fig 5). In the pre-treatment regimen, there was an increase in the enzymatic activity of SOD, GPx, GST and GR in the Cu-only exposed group (*P*<0.05), but pre-treatment with lysine did not affect enzymatic activity (*P*>0.05). The activity of CAT was significantly lower in the lysine pre-treatment group than in the Cu-only exposed group (*P*<0.001). The GSH level in the Cu-only exposed group decreased compared with the control group (*P*<0.05), but pre-treatment with lysine did not change the GSH levels compared with the Cu-only exposed control (*P*>0.05). In addition, no significant differences were observed in the gene expression of SOD, CAT, GR, GST, GPx, Nrf2 and Keap1b in the three pre-treatment test groups (*P*>0.05, Fig 5). In the co-treatment regimen, enterocytes exposed to Cu only showed increased levels of both gene expression and enzymatic activities of SOD, GPx, GST and GR, but co-treatment with lysine resulted in a decrease in enzymatic activity and gene expression levels (*P*<0.001, *P*<0.01 and *P*<0.05) (Fig 4 and Fig 5). GSH content was lower in the Cu-only exposed group than in the control (*P*<0.05), but co-treatment with lysine increased GSH levels (*P*<0.05). The gene expression of Nrf2 followed a similar pattern to that observed with SOD activity (*P*<0.05). The Keap1b m-RNA level was decreased in the Cu-only exposed group (*P*<0.05), but co-treatment with lysine significantly elevated its m-RNA level (*P*<0.001). However, the activity and m-RNA levels of CAT did not change in the three test groups (*P*>0.05). In the post-treatment regimen, the Cu-only exposed group showed an increase in the activities of SOD, CAT, GST and GPx compared with the unexposed control group, but post-treatment with lysine significantly decreased CAT and GST activities only (*P*<0.001 and *P*<0.01). The GR activity and GSH content exhibited the opposite pattern of CAT activity (*P*<0.05). The m-RNA levels of CAT, GPx and GST were increased by Cu exposure (*P*<0.05), but only GST gene expression was decreased by post-treatment with lysine (*P*<0.001). GR gene expression displayed the opposite pattern of GST gene expression (*P*<0.05). The Keap1b m-RNA level was decreased in the Cu-only exposed group (*P*<0.05), but post-treatment with lysine did not change its m-RNA level (*P*>0.05). However, no difference was observed in the gene expression of SOD or Nrf2 in the three test groups (*P*>0.05).

#### 3.5.4 Free Cu2+ concentration

We have determined the free Cu^2+^ concentration in the culture medium under co-treatment conditions (Table 3). The free Cu^2+^ concentrations in the control group were below the detection limit. Co-treatment with lysine significantly decreased the free Cu^2+^ concentrations in comparison to the Cu^2+^ control group (*P*<0.01).

**Table 3:** Effects of co-treatment of lysine on free Cu^2+^ ion concentrations (mg L^-1^) in medium of fish primary enterocytes cells^1^.

Treatments	Free Cu^2+^ ion concentrations
Ctrl+ctrl	--
Ctrl+Cu	2.18±0.08
Lys(180)+Cu	1.61±0.05**

^1^ The data represent the means±S.E. of four replicates.

*P<0.05

**P<0.01, and

***P<0.001 indicated significant difference from the 6 mg L^-1^ Cu alone group, with Student’s t-test.

## Discussion

Our study showed that fish fed a lysine deficient diet exhibited growth depression by the regulation of intestinal digestive and brush border enzyme activities and mRNA translation (supplementary data). It has been demonstrated that the integrity of the intestines is very important in fish growth, which is closely related to antioxidant ability [45]. Thus, we explored the effects of lysine on the intestinal antioxidant ability in young grass carp.

### 4.1 Lysine improved the antioxidant status in the intestine of fish

In fish, protein carbonyl (PC) content and malondialdehyde (MDA) content are commonly used as criteria of protein and lipid injury, respectively [46]. The results of this study showed that the MDA and PC contents were reduced in conjunction with increased levels of dietary lysine (up to a certain point), suggesting that intestinal lipid and protein damage are reduced by an optimal lysine concentration. In general, the protective ability of dietary lysine against oxidative damage may correlate with an increased antioxidant ability, which can be reflected by antioxidant enzymatic activities and non-enzymatic antioxidants [47]. In our study, an optimal dietary lysine concentration significantly increased the activities of CAT, SOD, GST, GPx, and GR, as well as the GSH level, suggesting that lysine possesses enzymatic and non-enzymatic antioxidant abilities in the intestine of fish. In rat, increases in antioxidant enzyme activities were partially related to an increase in their mRNA levels [3]. In our current study, the gene expression of SOD, CAT, GPx and GST were increased by dietary lysine to certain values, which followed the same trend as the enzymatic activities, suggesting that enhanced antioxidant enzyme activity may be closely related to the lysine up-regulation of gene transcription of antioxidant enzymes in fish. In addition, the signalling molecule Nrf2 and its suppressor Keap1 could modulate the antioxidant enzyme mRNA levels [23]. Our study was the first to show that an optimal dietary lysine concentration increased the mRNA level of Nrf2, but decreased the m-RNA level of Keap1b, whereas the Keap1a gene expression did not change as a result of the dietary lysine level. This indicates that lysine may elevate fish antioxidant enzyme mRNA levels through up-regulating Nrf2 gene transcription. However, to date, the effects of lysine on Nrf2 and Keap1 gene expression in fish have not been reported. Nutrient protection against the deleterious effects of reactive oxidants is conferred via multiple pathways: prevention, interception and repair [48]. Therefore, we established the fish primary enterocyte cell model to further explore the potential pathway by which lysine improves the fish antioxidant response.

### 4.2 Protection of lysine against Cu-induced oxidative damage in fish primary enterocytes

#### 4.2.1 Establishment of an optimal concentration of lysine against Cu-induced oxidative damage in an *in vitro* model

In this study, we used primary enterocytes to investigate the protective effect of lysine through pre-, co- and post-treatment regimens. CuSO_4_ was chosen as a pro-oxidant in this study because of its extensive use [49]. To our knowledge, the leakage of LDH is a well-known marker of metal-induced oxidative injury in cells [50]. Our study showed that the LDH activity significantly increased in the medium of the Cu-only exposed group, but the values decreased with pre-, co- and post-treatments with optimal concentrations of lysine, indicating that lysine protected the enterocytes against Cu-induced oxidative injury. Moreover, cellular growth is sensitive to Cu-induced oxidative damage, which is closely associated with cell viability and differentiation [51]. Studies have shown that MTT OD values and ALP activity are widely used to assess viability and differentiation in many cell types [52,53]. In our study, MTT OD values were significantly depressed in the Cu-only exposed group, but the values improved with increasing concentrations of lysine in pre-, co- and post-treatment group. The ALP activities in co- and post-treatment conditions showed the same pattern as the MTT OD values, indicating that lysine facilitated enterocyte growth. Based on these results, a medium with 180 mg lysine L^-1^ was used as the dose to further investigate the protective mechanism of lysine against Cu-induced oxidative damage.

#### 4.2.2 Lysine differentially alleviated Cu-induced lipid, protein and DNA oxidative damage *in vitro*

In this current study, lysine alleviated oxidative damage of lipids and proteins differentially. Among the pre-, co- and post-treatment regimens, co-treatment with lysine was found to have maximal ameliorative effects. Within the co- and post-treatment groups, Cu exposure induced an increase in the MDA and PC contents, but these values decreased in the medium with 180 mg lysine L^-1^, indicating that the co- and post-treatments with lysine might decrease Cu-induced lipid and protein damage in fish enterocytes cells. However, our study showed that 4-HNE content decreased in the co-treatment regimen but did not change in the post-treatment regimen. First, in comparison to the co-treatment regimen, there is a unique difference between these two Cu-only exposed groups. In co-treatment regimen, we used a lysine-free DMEM medium with 6 mg/L Cu for 24 h exposure, while the post-treatment regimen used commercial DMEM containing 146 mg/L of lysine HCL in the Cu-only exposed group. Therefore, the increased 4-HNE content in the co-treatment regimen might have resulted from excessive ROS production in the 24 h Cu exposure. Manabe et al. [54] reported that excessive amounts of ROS increased 4-HNE accumulation in human cells. Second, it is possible that 4HNE was quickly degraded after the blockage of Cu in the post-treatment group. In rat hepatocytes, 4-HNE (100 uM) is degraded completely within 3 min of incubation [55]. Interestingly, pre-treatment with lysine did not decrease MDA, PC or 4HNE contents, indicating that pre-treatment with lysine could not prevent Cu-induced lipid and protein damage in fish enterocytes. Taken together, this indicates that lysine may protect against Cu-induced oxidative damage in fish enterocytes under co- and post-treatment conditions. In general, the protective effects against oxidative damage may be partially related to the change in the antioxidant system. Thus, we further examined the antioxidant system to elucidate the mechanism of lysine protection against Cu-induced oxidative damage in the co- and post-treatment regimens.

#### 4.2.3 Lysine intercepted the Cu-induced oxidative damage in fish enterocytes

To our knowledge, Cu-induced oxidative damage to animals is mainly attributed to redox cycling between Cu^+^ and Cu^2+^, which induces ROS overproduction [56]. In our study, the free Cu^2+^ concentration in the culture medium of the group co-treated with lysine was lower than the Cu-exposure control group, suggesting that lysine may prevent Cu-induced oxidative damage by partially chelating Cu^2+^ in fish. In human serum albumin, lysine is responsible for Cu^2+^ binding [57]. The chelation effects may be ascribed to the functionality of the lysine amino group. Selvakannan et al. [58] reported that the lysine α-amino group could stabilize metal by forming hydrogen bonds between the lysine ɛ-amino group and the neighbouring carboxylic acid group, thereby decreasing free metal concentrations. Apart from this, the protection against Cu-induced oxidative damage may be related to the enzymatic antioxidant system that plays an important role in eliminating ROS [7]. In our studies, SOD, GPx, GST and GR activities were increased significantly in the Cu-only exposed alone group, but the levels decreased with lysine co-treatment compared with the Cu-only exposed group. These results suggest that lysine induced the adaptive antioxidant reaction. It is possible that the antioxidant enzyme levels are up-regulated in the early stages but decreased later when the oxidative damage is reduced by lysine co-treatment. Consistent with this hypothesis, Barrera et al. [59] found that the activity of CAT in rat kidneys at 24 h in the SnCl_2_ protective group was slightly higher than in the control, which significantly decreased at 48 h. Moreover, this may be partially associated with the regulation of antioxidant enzyme gene expression by lysine in fish. In aged rats, exercise training decreases MnSOD activity by regulating its mRNA level [60]. The results of the current study demonstrated that the mRNA levels of SOD, GPx, GST and GR were elevated in the Cu-only exposed group but significantly decreased in the lysine co-treatment group, which exhibited the same patterns as their respective enzyme changes. Positive correlations were observed between enzymatic activity and the gene expression of SOD (*r* = +0.996, *P* = 0.06), GPx (*r* = +0.987, *P* = 0.10) and GST (*r* = +0.978, *P* = 0.13), indicating that the change in antioxidant enzyme activity was partially attributed to the lysine-induced down-regulation of m-RNA levels of antioxidant protection genes in fish. However, the exact mechanism for this should be studied. Additionally, antioxidant enzyme gene expression could be mediated by modification of the signalling molecule Nrf2 [61]. In our study, Nrf2 gene expression was increased in the Cu-only exposed group but significantly decreased in the lysine co-treatment group. Lysine-mediated decreases in Nrf2 expression may be related to the promotion of Nrf2 degradation. McMahon et al. [62] reported that Keap1 has been identified as an Nrf2-binding protein, which facilitates Nrf2 degradation. In our study, Keap1b gene expression was increased by co-treatment with lysine, suggesting that lysine may increase Keap1b m-RNA levels to promote Nrf2 degradation. Lo et al. [63] reported that seven lysine residues located in Nrf2 play an important role in Keap1-dependent degradation. Interestingly, our *in vivo* study indicated that optimal lysine concentration elevated Nrf2 expression in the intestine of grass carp. The different results may be attributed to the chelation effects of lysine. Wang et al. [64] reported that copper could also mediate the activation of the Nrf2 signalling pathway in human cells. Therefore, the reduction of Nrf2 by lysine co-treatment *in vitro* may be related to the chelation of lysine and Cu, which offsets activation effects. Future studies should test this hypothesis. Interestingly, our study found that the activity and gene expression of CAT, a downstream Nrf2 target, did not correlate with Nrf2 upregulation. This imbalance could be attributed to two reasons. First, there is a discrepancy between the gene expression of Nrf2 and its downstream target antioxidant enzyme in some circumstances. Wang et al. [65] reported that the gene expression of CAT was not increased following the upregulation of Nrf2 in the serum of mice. Second, CAT activity could be inhibited by superoxide produced by Cu exposure [66].

#### 4.2.4 Lysine promoted enterocyte repair after Cu challenge

The living cell is constantly exposed to potential oxidants, and damage occurs continuously [67]. Therefore, the repair system is very important for coping with stress and damage [13]. To our knowledge, antioxidant enzymes and non-enzymatic antioxidants play an important role in mammalian oxidative damage repair [68,69]. Schafer and Buettner [70] found that in humans, cellular GSH could repair protein sulfhydryl groups directly by reversible self-oxidation. In our study, Cu alone decreased the GSH content, but post-treatment with lysine could increase the GSH level compared with the Cu-only exposed control. This finding suggests that lysine post-treatment may elevate the GSH level to repair oxidative damage proteins directly. This may be due to lysine promoting the de novo synthesis of GSH in fish. In rainbow trout liver, lysine is catabolised by the saccharopine pathway, in which glutamate is produced [19]. Stoll et al. [20] reported that enteral glutamate can be converted into GSH in piglets. Another possibility may be that lysine facilitates GSH re-reduction. Kohen and Nyska [71] reported that the glutathione reductase (GR) enzyme is involved in GSH re-reduction in biological systems. In our study, GR activity and gene expression were increased by post-treatment with lysine, indicating that lysine may promote GSH re-reduction by GR.

Moreover, the repair capacity is closely related to the cellular redox state of humans [72]. Matés [73] reported that the redox state of a cell is extremely sensitive to antioxidant enzyme activities. In our study, significant increases in CAT and GST activities were observed in the Cu-only exposed group, but the level decreased with lysine supplementation, suggesting that lysine might improve enterocyte repair by the modulation of antioxidant enzyme activities. However, the gene expression of Nrf2, Keap1, and its downstream target enzymes (SOD and CAT) did not change in the lysine post-treatment group. Similar observations were made in the liver of rats, which showed that vitamin E deficiency did not activate Nrf2 or Nrf2-mediated antioxidant enzymes [74]. Taken together, this demonstrates that post-treatment with lysine may exhibit post-transcriptional effects on antioxidant enzyme, which is different from what was observed in the co-treatment group. However, the detailed mechanism in fish requires further investigation.

Apart from these mechanisms, oxidative damage proteins could also be removed and degraded directly [13]. Davies [75] reported that oxidised proteins are recognized and degraded by the proteasome complex. In our study, proteasome activity was significantly decreased in the Cu-only exposed alone group, but post-treatment with lysine elevated proteasome activity compared with the Cu-only exposed group. This suggests that lysine may promote the removal of damaged proteins by increasing the activity of the proteasome complex. Saeki et al. [76] reported that lysine-linked ubiquitin chains served as the proteasomal degradation signal in yeast. However, the exact mechanism in fish deserves further investigation. Moreover, the damage can also be repaired at the DNA level directly [75]. 8-hydroxydeoxyguanosine (8OHdG) is a biomarker of oxidative DNA damage in mouse [77]. In our study, the 8OHdG level was decreased in the post-treatment lysine group, indicating that lysine post-treatment repaired DNA damage. Studies have shown that acetylation of key protein lysine residue play an important role in the repair of damaged DNA [78]. Kim et al. [79] reported that dietary components have been found to modulate lysine acetylation levels *in vivo*. However, whether lysine can affect DNA repair by changing the lysine acetylation levels in fish is unknown. In conclusion, we have demonstrated a novel role for lysine in protecting enterocytes against Cu-induced oxidative damage. The potential action pathway is presented in S2 Fig.

## Supporting Information

S1 Fig Relative mRNA abundance of intestinal copper/zinc superoxide dismutase (SOD), catalase (CAT), glutathione peroxidase (GPx), glutathione reduces (GR), glutathione-S-transferase (GST), NF-E2-related factor-2 (Nrf2), Kelch-like ECH-associated protein 1a (Keap1a) and Kelch-like ECH-associated protein 1b (Keap1b) of fish fed the experimental diets. (ZIP)

S2 Fig Schema illustrating protective action pathway of lysine on Cu-induced oxidative damage in fish enterocytes. (TIF)

S1 Table Formulation and nutritional parameter of basal diets. (DOCX)

S2 Table Real-time quantitative PCR primers. (DOCX)

S3 Table Relative mRNA abundance of digestive enzyme (tryspinogen1, chymotrypsinogen and amylase) gene in intestine, brush border enzyme (CK and Na^+^, K^+^-ATPase), TOR and 4E-BP gene in proximal intestine (PI), mid intestine (MI) and distal intestine (DI) of fish fed the experimental diets for 56 days. (DOCX)

S4 Table Effects of pre-, co- and post-treatment of lysine on the lactic acid dehydrogenase (LDH, U/g prot) activity, 3-(4, 5-dimethylthiazol-2-yl)-2,5-diphenyltetrazolium bromide (MTT) OD and alkaline phosphatase (ALP, mmol of nitrophenol released g^-1^ tissue h^-1^) activity of fish primary enterocytes cells. (DOCX)
