# Supplementary figures and images for: Co- and Post-Treatment with Lysine Protects Primary Fish Enterocytes against Cu-Induced Oxidative Damage
Source: PLoS One. 2016 Jan 26;11(1):e0147408. doi: 10.1371/journal.pone.0147408 (PMC4727818; doi:10.1371/journal.pone.0147408)

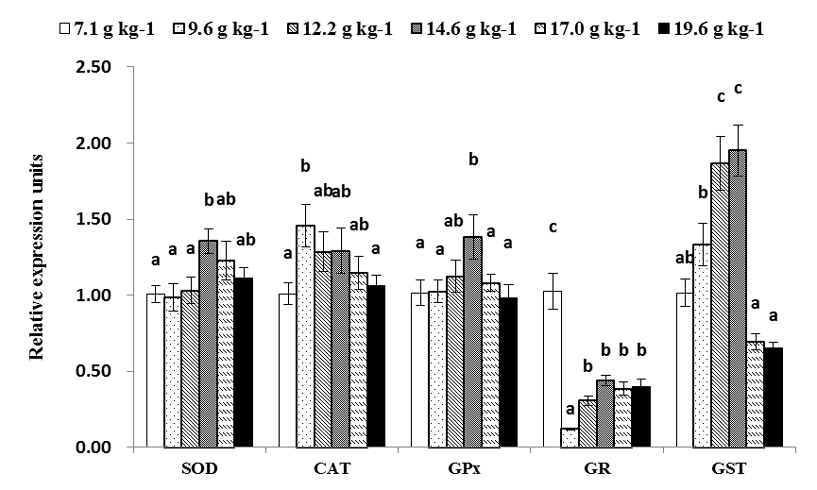

Supplement: S1 Fig — (ZIP) [file pone.0147408.s001.zip › S1 Fig/S1 Fig a.tif]

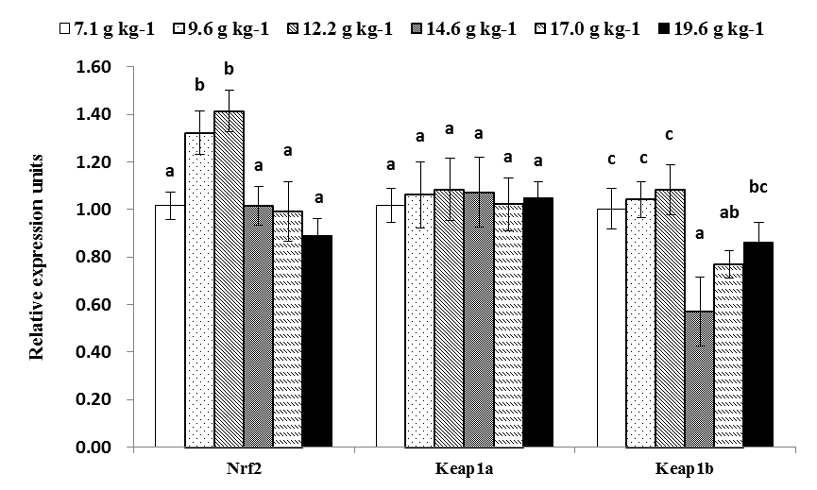

Supplement: S1 Fig — (ZIP) [file pone.0147408.s001.zip › S1 Fig/S1 Fig b.tif]

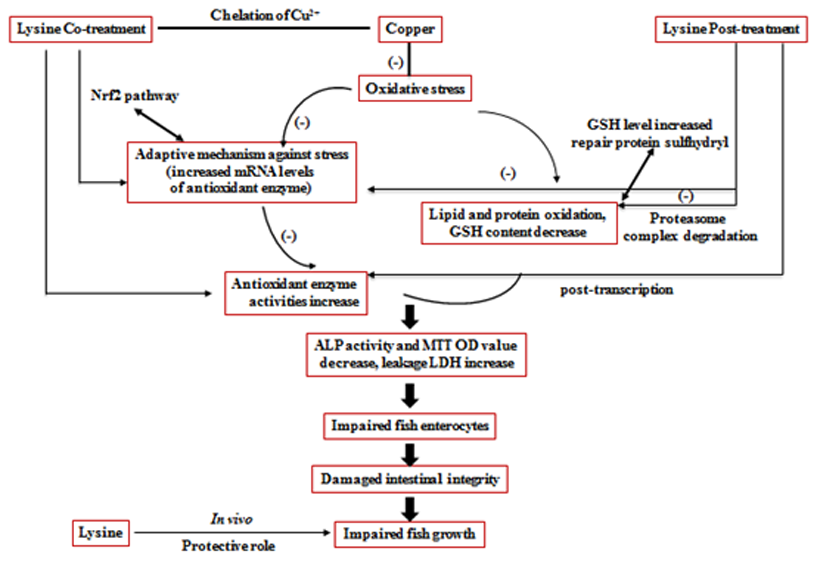

Supplement: S2 Fig — (TIF) [file pone.0147408.s002.tif]
