# Supplementary material for: Co- and Post-Treatment with Lysine Protects Primary Fish Enterocytes against Cu-Induced Oxidative Damage
Source: PLoS One. 2016 Jan 26;11(1):e0147408. doi: 10.1371/journal.pone.0147408 (PMC4727818; doi:10.1371/journal.pone.0147408)
Supplement: S1 Table — (DOCX) [file pone.0147408.s003.docx]

**S1 Table**

Formulation and nutritional parameter of basal diets

| Ingredients | g kg ^-1^ | Nutrients content^1^ | g kg ^-1^ |
| --- | --- | --- | --- |
| Fish meal | 78.0 | Crude protein | 295.4 |
| Casein | 30.0 | Crude lipid | 46.8 |
| Gelatin | 39.9 | ω-3 | 10.0 |
| Amino acids mix^2^ | 149.9 | ω-6 | 10.0 |
| Lysine premix^3^ | 30.0 | Lysine | 7.1 |
| Glycine premix^4^ | 79.7 | Available phosphorus | 6.0 |
| Corn starch | 92.4 |  |  |
| α-starch | 300.0 |  |  |
| Fish oil | 22.0 |  |  |
| Soya bean oil | 18.9 |  |  |
| Trace mineral premix^5^ | 20.0 |  |  |
| vitamin premix^6^ | 10.0 |  |  |
| Ca(H_2_PO_4_)_2_ | 22.7 |  |  |
| Choline chloride | 6.0 |  |  |
| α-cellulose | 100.0 |  |  |
| Ethoxyquin | 0.5 |  |  |

^1^Crude protein and crude lipid were measured value. Available phosphorus, n-3 and n-6 contents were calculated according to NRC (1993)

^2^Amino acid mix (g kg^-1^): arginine, 12.7; histidine, 7.9; isoleucine, 12.6; leucine, 20.4; methionine, 7.8; cystine, 0.91; phenylalanine, 13.5; tyrosine, 10.8; threonine, 12.1; tryptophan, 3.57; valine, 15.3; glutamic acid, 32.3

^3^L-lysine sulphate was added to obtain graded level of lysine. Per kilogram of lysine premix composition from diet 1 to 6 was as follows (g kg^-1^): L-lysine sulphate 0.00, 141.00, 282.00, 423.01, 564.02, 705.02 g and corn starch 1000, 859.00, 718.00, 576.99, 435.98, 294.98 g, respectively.

^4^Each mixture was made isonitrogenous with addition of reduced amounts of glycine and compensated with appropriate amounts of corn starch. Per kilogram of glycine premix composition from diet 1 to 6 was as follows (g kg^-1^): glycine 788.09, 755.88, 723.66, 691.45, 659.23, 627.02 g and corn starch 211.91, 244.12, 276.34, 308.55, 340.77, 372.98 g, respectively.

^5^Per kilogram of mineral premix (g kg^-1^): FeSO_4_·H_2_O, 25.00 g; CuSO_4_·5H_2_O, 0.60 g; ZnSO_4_·H_2_O, 4.35 g; MnSO_4_·H_2_O, 2.04 g; KI, 1.10 g; NaSeO_3_, 2.50 g; MgSO_4_·H_2_O, 230.67 g; corn starch 733.74 g. All ingredients were diluted with corn starch to 1 kg.

^6^Per kilogram of vitamin premix (g kg^-1^): retinyl acetate (5000,00 IU g^-1^), 0.80 g; cholecalciferol (5000,00 IU g^-1^), 0.48 g; DL-α-tocopherol acetate (500 g kg^-1^), 20.00 g; menadione (230 g kg^-1^), 0.22 g; thiamine hydrochloride (980 g kg^-1^), 0.12 g; riboflavin (800 g kg^-1^), 0.99 g; pyridoxine hydrochloride (980 g kg^-1^), 0.62 g; cyanocobalamin (10 g kg^-1^), 0.10 g; niacin (990 g kg^-1^), 2.58 g; D-biotin (20 g kg^-1^), 5.00 g; meso-inositol (990 g kg^-1^), 52.33 g; folic acid (960 g kg^-1^), 0.52 g; ascorhyl acetate (930 g kg^-1^), 7.16 g; calcium-D-pantothenate (900 g kg^-1^), 2.78 g. All ingredients were diluted with corn starch to 1 kg
