# Supplementary material for: Co- and Post-Treatment with Lysine Protects Primary Fish Enterocytes against Cu-Induced Oxidative Damage
Source: PLoS One. 2016 Jan 26;11(1):e0147408. doi: 10.1371/journal.pone.0147408 (PMC4727818; doi:10.1371/journal.pone.0147408)
Supplement: S2 Table — (DOCX) [file pone.0147408.s004.docx]

**S2 Table**

Real-time quantitative PCR primers.

| Gene |  | Primers | Annealing temperature (℃) | Accession number |
| --- | --- | --- | --- | --- |
| Trypsinogen1 | F | 5′- CTGCTGCTCACTGCTACAA-3′ | 59.3 | JX854450 |
|  | R | 5′- TACTGCTCAGAACCCTCATT-3′ |  |  |
| chymotrypsinogen | F | 5′-GGAAAGTCCATCATACACCC-3′ | 60.4 | JX854443 |
|  | R | 5′-AGCCTCCAGCGAAGTTG-3′ |  |  |
| Na^+^/K^+^-ATPase | F | 5′-TGCCATTGTAGCCGTAAC-3′ | 60.3 | JX854442 |
|  | R | 5′-GGTGCCCAAAGGTAGAGG-3′ |  |  |
| Amylase | F | 5′-ACTATGTGCGTGGTAAGGT-3′ | 57.1 | FJ641975.1 |
|  | R | 5′-CTTGATGTAATAGGCTCCC-3′ |  |  |
| Creatine kinase | F | 5′-CTCCTCGTTCACCCAGAC-3′ | 61.4 | JX854444 |
|  | R | 5′-CAGCATCAAGGGATACGC-3′ |  |  |
| TOR | F | 5′-TCCCACTTTCCACCAACT-3′ | 61.4 | JX854449 |
|  | R | 5′-ACACCTCCACCTTCTCCA-3′ |  |  |
| 4E-BP | F | 5′-TTTCTACAAGCCAAGCCAC-3′ | 55.0 | JX854451 |
|  | R | 5′-CAACCATGATGCCAAACC-3′ |  |  |
| SOD | F | 5′-CGCACTTCAACCCTTACA-3′ | 61.5 | GU901214 |
|  | R | 5′-ACTTTCCTCATTGCCTCC-3′ |  |  |
| GPx | F | 5′-GGGCTGGTTATTCTGGGC-3′ | 61.5 | EU828796 |
|  | R | 5′-AGGCGATGTCATTCCTGTTC-3′ |  |  |
| CAT | F | 5′-GAAGTTCTACACCGATGAGG-3′ | 58.7 | FJ560431 |
|  | R | 5′-CCAGAAATCCCAAACCAT-3′ |  |  |
| GR | F | 5′-GTGTCCAACTTCTCCTGTG-3′ | 59.4 | JX854448 |
|  | R | 5′-ACTCTGGGGTCCAAAACG-3′ |  |  |
| GST | F | 5′-TCTCAAGGAACCCGTCTG-3′ | 58.4 | EU107283 |
|  | R | 5′-CCAAGTATCCGTCCCACA-3′ |  |  |
| Nrf2 | F | 5′-CTGGACGAGGAGACTGGA-3′ | 62.5 | KF733814 |
|  | R | 5′-ATCTGTGGTAGGTGGAAC-3′ |  |  |
| Keap1a | F | 5′-TTCCACGCCCTCCTCAA-3′ | 63.0 | KF811013 |
|  | R | 5′-TGTACCCTCCCGCTATG-3′ |  |  |
| Keap1b | F | 5′-TCTGCTGTATGCGGTGGGC-3′ | 57.9 | KJ729125 |
|  | R | 5′-CTCCTCCATTCATCTTTCTCG-3′ |  |  |
| β-Actin | F | 5′-GGCTGTGCTGTCCCTGTA-3′ | 61.4 | M25013 |
|  | R | 5′-GGGCATAACCCTCGTAGAT-3′ |  |  |
