# Supplementary material for: Co- and Post-Treatment with Lysine Protects Primary Fish Enterocytes against Cu-Induced Oxidative Damage
Source: PLoS One. 2016 Jan 26;11(1):e0147408. doi: 10.1371/journal.pone.0147408 (PMC4727818; doi:10.1371/journal.pone.0147408)
Supplement: S3 Table — (DOCX) [file pone.0147408.s005.docx]

**S3 Table**

Relative mRNA abundance of digestive enzyme (tryspinogen1, chymotrypsinogen and amylase) gene in intestine, brush border enzyme (CK and Na^+^, K^+^-ATPase), TOR and 4E-BP gene in proximal intestine (PI), mid intestine (MI) and distal intestine (DI) of fish fed the experimental diets for 56 days^1^

| Dietary lysine levels (g kg^-1^ diet) | | | | | | |
| --- | --- | --- | --- | --- | --- | --- |
|  | 7.1 | 9.6 | 12.2 | 14.6 | 17.0 | 19.6 |
| Trypsinogen1 | 1.01±0.08^a^ | 0.99±0.07^a^ | 1.39±0.17^b^ | 1.36±0.16^b^ | 0.93±0.07^a^ | 0.96±0.08^a^ |
| Choymotrypsinogen | 1.02±0.10^ab^ | 1.26±0.11^b^ | 1.06±0.06^ab^ | 1.09±0.14^ab^ | 0.87±0.10^a^ | 0.80±0.06^a^ |
| Amylase | 1.03±0.10^a^ | 1.06±0.06^a^ | 1.09±0.08^a^ | 1.06±0.04^a^ | 1.05±0.06^a^ | 1.02±0.09^a^ |
| Na^+^/K^+^-ATPase |  | | | | | |
| PI | 1.01±0.01^ab^ | 1.32±0.06^bc^ | 1.52±0.08^c^ | 0.89±0.04^a^ | 0.85±0.06^a^ | 0.82±0.09^a^ |
| MI | 1.02±0.11^b^ | 1.07±0.07^b^ | 1.34±0.12^c^ | 0.89±0.06^ab^ | 0.88±0.07^ab^ | 0.64±0.07^a^ |
| DI | 1.01±0.09^ab^ | 1.20±0.16^ab^ | 1.38±0.18^b^ | 1.00±0.08^ab^ | 0.95±0.11^a^ | 0.96±0.12^a^ |
| CK |  | | | | | |
| PI | 1.02±0.11^a^ | 1.10±0.14^a^ | 1.47±0.16^a^ | 1.42±0.16^a^ | 1.36±0.16^a^ | 1.41±0.10^a^ |
| MI | 1.02±0.12^a^ | 0.99±0.10^a^ | 1.05±0.10^a^ | 1.58±0.13^b^ | 1.49±0.15^b^ | 1.41±0.08^b^ |
| DI | 1.02±0.12^a^ | 1.07±0.08^a^ | 1.09±0.08^a^ | 1.32±0.17^a^ | 1.33±0.12^a^ | 1.37±0.10^a^ |
| TOR |  |  |  |  |  |  |
| PI | 1.10±0.09^b^ | 1.05±0.01^b^ | 1.11±0.11^b^ | 1.16±0.13^b^ | 1.54±0.14^c^ | 0.72±0.05^a^ |
| MI | 0.94±0.10^a^ | 0.96±0.05^a^ | 1.38±0.14^bc^ | 1.56±0.11^c^ | 1.19±0.09^ab^ | 1.21±0.13^ab^ |
| DI | 1.03±0.11^a^ | 1.20±0.03^ab^ | 1.78±0.15^c^ | 1.42±0.15^b^ | 1.10±0.04^ab^ | 1.19±0.14^ab^ |
| 4E-BPs |  |  |  |  |  |  |
| PI | 1.09±0.10^a^ | 0.92±0.08^a^ | 0.92±0.09^a^ | 0.93±0.04^a^ | 1.19±0.12^a^ | 1.15±0.11^a^ |
| MI | 1.17±0.05^a^ | 0.99±0.12^a^ | 0.90±0.07^a^ | 1.05±0.10^a^ | 1.04±0.11^a^ | 1.08±0.04^a^ |
| DI | 1.17±0.11^bc^ | 1.23±0.13^bc^ | 0.86±0.05^a^ | 1.01±0.12^ab^ | 0.98±0.06^ab^ | 1.32±0.10^c^ |

^1^Results are the means ± SE (n= 5). Different letter within the same row denoted significant differences (*P*<0.05), with Duncan's multiple range tests.
