# Supplementary material for: Co- and Post-Treatment with Lysine Protects Primary Fish Enterocytes against Cu-Induced Oxidative Damage
Source: PLoS One. 2016 Jan 26;11(1):e0147408. doi: 10.1371/journal.pone.0147408 (PMC4727818; doi:10.1371/journal.pone.0147408)
Supplement: S4 Table — (DOCX) [file pone.0147408.s006.docx]

**S4 Table**

Effects of pre-, co- and post-treatment of lysine on the lactic acid dehydrogenase (LDH, U/g prot) activity, 3-(4, 5-dimethylthiazol-2-yl)-2,5-diphenyltetrazolium bromide (MTT) OD and alkaline phosphatase (ALP, mmol of nitrophenol released g^-1^ tissue h^-1^) activity of fish primary enterocytes cells^1^.

| Treatments | Ctrl+ctrl | Ctrl+Cu | Lys(60)+Cu | Lys(120)+Cu | Lys(180)+Cu | Lys(240)+Cu | Lys(300)+Cu |
| --- | --- | --- | --- | --- | --- | --- | --- |
| MTT |  |  |  |  |  |  |  |
| Pre-treatment | 0.123±0.005 | 0.111±0.003^#^ | 0.117±0.004 | 0.122±0.001^**^ | 0.126±0.002^***^ | 0.135±0.003^***^ | 0.134±0.003^***^ |
| Co-treatment | 0.140±0.003 | 0.127±0.003^#^ | 0.145±0.002^**^ | 0.144±0.002^**^ | 0.155±0.004^**^ | 0.154±0.003^**^ | 0.157±0.003^**^ |
| Post-treatment | 0.128±0.003 | 0.115±0.001^#^ | 0.121±0.003 | 0.127±0.001^**^ | 0.137±0.002^***^ | 0.140±0.002^***^ | 0.137±0.004^**^ |
| LDH |  |  |  |  |  |  |  |
| Pre-treatment | 26.01±0.26 | 42.21±1.14^#^ | 37.38±1.05^*^ | 36.14±1.08^*^ | 35.05±1.40^**^ | 32.81±1.26^**^ | 33.14±1.15^**^ |
| Co-treatment | 29.15±0.05 | 35.61±0.81^#^ | 34.28±0.46 | 33.48±1.60 | 30.69±0.45^**^ | 30.33±0.48^**^ | 29.57±1.90^**^ |
| Post-treatment | 15.35±0.55 | 36.38±1.55^#^ | 36.91±1.37 | 31.28±0.71^*^ | 28.17±1.38^**^ | 28.96±1.31^*^ | 27.22±1.17^**^ |
| ALP |  |  |  |  |  |  |  |
| Pre-treatment | 3.81±0.19 | 2.94±0.13^#^ | 3.00±0.11 | 3.11±0.07 | 3.04±0.06 | 3.02±0.12 | 3.04±0.11 |
| Co-treatment | 3.67±0.12 | 2.17±0.05^#^ | 2.22±0.02 | 2.80±0.08^**^ | 3.68±0.08^***^ | 3.85±0.08^***^ | 3.82±0.05*** |
| Post-treatment | 4.13±0.12 | 3.03±0.08^#^ | 3.09±0.11 | 3.34±0.10 | 3.59±0.04^**^ | 3.65±0.16^*^ | 3.55±0.12* |

^1^The data represent the means±S.E. of four replicates. # indicated significant difference compared with control values, and ^*^*P*<0.05, ^**^*P*<0.01, and ^***^*P*<0.001 indicated significant difference from the 6 mg L^-1^ Cu alone group, with Student’s t-test.
